# Nicotine Exacerbates TAAD Formation Induced by Smooth Muscle-Specific Deletion of the TGF-*β* Receptor 2

**DOI:** 10.1155/2021/6880036

**Published:** 2021-10-01

**Authors:** Changzoon Chun, Xiaoyan Qi, Fen Wang, Kyle B. Madrid, Lennon A. Saldarriaga, Max R. Fisch, Mark L. Brantly, Gilbert R. Upchurch, Zhihua Jiang

**Affiliations:** ^1^Division of Vascular Surgery and Endovascular Therapy, University of Florida College of Medicine, Gainesville, FL 32603, USA; ^2^Department of Medicine, University of Florida College of Medicine, Gainesville, FL 32603, USA

## Abstract

Tobacco smoke is an established risk factor for thoracic aortic aneurysms and dissections (TAAD). However, little is known about its underlying mechanisms due to the lack of validated animal models. The present study developed a mouse model that may be utilized to investigate exacerbation of TAAD formation by mimetics of tobacco smoke. TAADs were created via inducible deletion of smooth muscle cell-specific *Tgfbr2* receptors. Using this model, the first set of experiments evaluated the efficacy of nicotine salt (34.0 mg/kg/day), nicotine free base (NFB, 5.0 mg 90-day pellets), and cigarette smoke extract (0.1 ml/mouse/day). Compared with their respective control groups, only NFB pellets promoted TAAD dilation (23 ± 3% vs. 12 ± 2%, *P* = 0.014), and this efficacy was achieved at a cost of >50% acute mortality. Infusion of NFB with osmotic minipumps at extremely high, but nonlethal, doses (15.0 or 45.0 mg/kg/day) failed to accelerate TAAD dilation. Interestingly, costimulation with *β*-aminopropionitrile (BAPN) promoted TAAD dilation and aortic rupture at dosages of 3.0 and 45.0 mg/kg/day, respectively, indicating that BAPN sensitizes the response of TAADs to NFB. In subsequent analyses, the detrimental effects of NFB were associated with clustering of macrophages, neutrophils, and T-cells in areas with structural destruction, enhanced matrix metalloproteinase- (MMP-) 2 production, and pathological angiogenesis with attenuated fibrosis in the adventitia. In conclusion, modeling nicotine exacerbation of TAAD formation requires optimization of chemical form, route of delivery, and dosage of the drug as well as the pathologic complexity of TAADs. Under the optimized conditions of the present study, chronic inflammation and adventitial mal-remodeling serve as critical pathways through which NFB exacerbates TAAD formation.

## 1. Introduction

Thoracic aortic aneurysms and dissections (TAADs) are a silent killer. Clinical diagnosis of TAADs prior to complications is notoriously difficult [1]. Only 5% of TAADs are symptomatic prior to dissection or rupture, and among those with symptoms, fewer than 50% may be correctly diagnosed in the emergency room [2]. One of the major risk factors for TAAD development is smoking [3, 4]. A recent prospective study with age- and sex-adjusted analysis identified smoking as an independent risk factor for the development of TAADs [5]. Additionally, smoking is associated with a several-fold higher incidence of abdominal aortic aneurysms (AAAs) and cerebral aneurysms [6–9]. Nicotine is a major active ingredient of e-cigarettes and tobacco smoke. Because the use of tobacco-related materials particularly (e)-cigarettes has been constantly increasing in the United States and other countries [10, 11], the number of TAAD cases will likely grow exponentially in the coming years, so the need to develop effective medical therapies is emerging.

Although smoking is a well-established risk factor for the development of AAAs and TAADs, current understanding of its underlying mechanisms remains elusive, with specific details yet to be revealed [12]. In the case of AAA formation, proteases, such as the matrix metalloproteinases MMP2 and MMP9, as well as elastase are elaborated by smooth muscle cells (SMCs), endothelial cells, and inflammatory cells of AAAs upon nicotine treatment [13]. Regulation of microRNA expression [14], production of ROS (radical oxygen species) [15], and epigenetic modification of immune cell phenotypes [9] have also been implicated as mechanisms underlying the detrimental effects of tobacco smoke on AAA formation. However, knowledge is scarce as to how tobacco smoke exacerbates the development of TAADs because of the lack of animal models. To meet this critical need, the present study developed a mouse model in which the phenotypic expression of TAADs can be modulated by tobacco smoke or its major component(s).

The development of TAADs differs in many aspects from AAAs, including the etiology and underlying mechanisms [16]. Strategies successfully applied to create AAAs responsive to tobacco smoke may not be transferable to studies focused on TAADs. Recent advances in human genetics have led to the creation of several mouse TAAD models that carry the pathogenic alleles identified in TAAD patients [17]. However, these models are not ideal for studying sporadic TAADs. Firstly, TAADs in these models rarely rupture. Additionally, the pathogenic alleles carried by these models are not present in the vast majority (~75%) of human TAAD cases, the so-called sporadic TAADs [16, 17]. Therefore, a model wherein the development of TAADs is not driven by germ line mutations would be more relevant to sporadic TAAD formation. Our laboratory has previously reported that deletion of smooth muscle cell-specific *Tgfbr2* (transforming growth factor type II receptor, *Tgfbr2^iko^*) induces spontaneous TAAD formation in adult mice, with the phenotype less severe than that expressed in *Tgfbr1^iko^* mice [18]. A study from another group also documented relatively low penetrance of *Tgfbr2^iko^* in adult mice [19]. The mild aortopathy induced by *Tgfbr2^iko^* provides a favorable baseline pathology for studies to evaluate the effects of risk factors that exacerbate TAAD development. With this model, the present study tested several protocols that had been successfully applied by other groups to model the impact of smoking in various diseases [20], including AAAs [14, 15]. Through optimization of multiple factors, a working protocol was established for nicotine exacerbation of TAAD formation in *Tgfbr2^iko^* mice. Mechanistic insights into the inflammatory response and adventitial remodeling that were obtained will help to understand the detrimental effects of nicotine on TAAD formation.

## 2. Results

### 2.1. Nicotine Salt Has Minimum Impact on Dilation of the TAADs Induced by *Tgfbr2^iko^*

Nicotine has been administered in various chemical formats and via different delivery approaches in published studies [20]. Osmotic pumps were utilized in this study to deliver nicotine at a constant and precise dose. Due to concerns about degradation of nicotine free base (NFB) at physiological pH conditions [21], nicotine bitartrate salt (NS) was used in initial experiments. Ultrasound imaging was performed to monitor dilation of the TAADs. Judging by aortic dilation, experiments throughout this study were terminated for gross evaluation and sample collection when a follow-up period longer than 28 days was determined to be uninformative. In cases when follow-up extended beyond 28 days, the implanted pumps were replaced with freshly prepared pumps on day 28.

**Figure 1:**
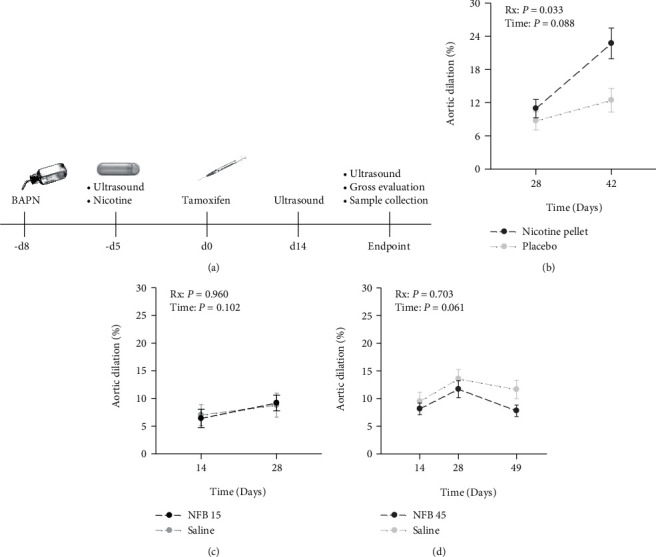
Nicotine free base (NFB) exacerbates TAAD formation only at a lethal dosage. (a) Experimental timeline schematic for drug administration and procedures. (b) Aortic dilation of mice that survived the initial burst of NFB release (*n* = 7). Control mice (*n* = 10) were implanted with placebo pellets. (c, d) Aortic dilation of mice infused with NFB at a rate of 15.0 (NFB 15) or 45.0 (NFB 45) mg/kg/day or saline (*n* = 8‐9 per group). Data were analyzed using two-way repeated measures ANOVA.

Mice (*Tgfbr2^f/f^*, *Mhy11-CreER^+^*, male, 9-10 weeks of age) were randomly assigned to two groups. Osmotic minipumps were implanted on -d5 to deliver saline (*n* = 9) or NS (34.0 mg/kg/day, equivalent to 11.1 mg/kg/day of NFB, *n* = 10). Tamoxifen (2.5 mg/day) was administered daily via *i.p.* injections for five consecutive days. The day that the first dose of tamoxifen was given was counted as day 0 (d0). Ultrasound imaging was performed every two to four weeks, beginning on d0 to record the baseline diameter of the TAADs and suprarenal aortas (SRAs). The same schedule was followed for all *in vivo* experiments performed throughout this study (Figure 1(a)). Mice included in this experiment were followed with ultrasound imaging. Because of the nearly complete overlapping in growth trajectory for both the TAADs (Rx: *P* = 0.624; time: *P* = 0.873; two-way RM ANOVA, Figure S1a) and the AAAs (data not shown) of mice treated with saline or nicotine salt, the experiment was terminated on d42. Representative ultrasound images obtained on d42 for each group are provided in Figure S1b.

### 2.2. Nicotine Free Base Alone Cannot Enhance Dilation of TAADs, except at a Lethal Dosage

Although nicotine salt has been successfully used in studies in the fields of neuroscience, cancer biology, and respiratory diseases [20], it appears that nicotine free base (NFB) is the preferred chemical format for studies focusing on AAA formation [14, 15]. We evaluated the efficacy of NFB delivered via nicotine pellets (NPs) at the dosage (5.0 mg, 90-day release, approximately 3.0 mg/kg/day) reported by Maegdefessel et al. [14]. Male mice, 9 to 17 weeks, were randomized to receive nicotine (*n* = 10) or placebo (*n* = 10) pellets. To our surprise, 5 of the 10 mice died shortly (within minutes to hours) after receiving NPs, while those given placebos all survived. Another cohort (10-17 weeks of age, *n* = 11) was then added to the NP group to increase the sample size. In this cohort, 9 mice died within a few hours. Interestingly, those that had survived the early lethal period later exhibited normal behavior and had body weights as well as blood pressures similar to those receiving placebo pellets (Figure S2a and S2b). TAADs of these survivors exhibited a significantly accelerated dilation compared with those treated with placebo (*P* = 0.033 by two-way RM ANOVA, Figure 1(b)). It appears that aging from 9 weeks to 17 weeks had minimum impacts on aortic diameter at the baseline (Figure S2c) and aortic dilation after treatment (Figure S2d).

Inspired by the outcome of NP-implanted survivor mice, we examined whether a nonlethal dosage of NFB is effective to promote TAAD formation. Mice (male, 13-18 weeks of age) were randomized to receive saline (*n* = 8) or NFB (15.0 mg/kg/day, *n* = 9). Animals in both groups all survived to the endpoint, with similar body weight gains (2.6 ± 0.4 g vs 2.6 ± 0.2, *P* = 1.0) during the four-week follow-up period. Compared to the saline controls, infusion of NFB at a rate of 15.0 mg/kg/day was unable to stimulate TAAD dilation (Figure 1(c)).

In a separate experiment, the dose of NFB was further increased to 45.0 mg/kg/day. Male mice (10-18 weeks of age) were randomly assigned to receive saline (*n* = 8) or NFB (*n* = 9) and followed with ultrasound imaging for 49 days. All animals survived until endpoint assessment and gained a similar amount of body weight as the control mice (2.4 ± 0.3 g and 3.3 ± 0.6 g for those treated with NFB and saline, respectively, *P* = 0.131). The concentration of cotinine in urine collected on d49 was >2,000 ng/ml for all NFB-treated mice, while it was not detectable in saline-treated controls. Despite the exposure to such an extremely high dosage of NFB, TAADs of the treated mice had advanced at a similar rate and were dilated to a similar degree as the controls by d49 (Figure 1(d)).

### 2.3. Cigarette Smoke Extract (CSE) Has a Moderate Impact on TAAD Dilation

The insufficient efficacy of administering pure NFB led us to develop alternative approaches. Among the various kinds of tobacco smoke mimetics, cigarette smoke extract (CSE) is another agent widely accepted for use in research [22]. We speculated that CSE may be more effective in modulating the aortic phenotype than nicotine alone, due to its complex ingredient composition. Male mice (8-15 weeks of age) were injected i.p. (0.1 ml, daily, 5 days a week) with CSE (*n* = 9) or PBS (*n* = 6). Ultrasound imaging revealed a trend of more severe TAAD dilation in mice treated with CSE than those treated with PBS. However, the average difference was quite small (5%) at d14 and stayed the same over the following two weeks (Figure S3). In view of this observation, the experiments were terminated at d28.

### 2.4. Cotreatment with BAPN Sensitizes the TAAD Response to NFB

**Figure 2:**
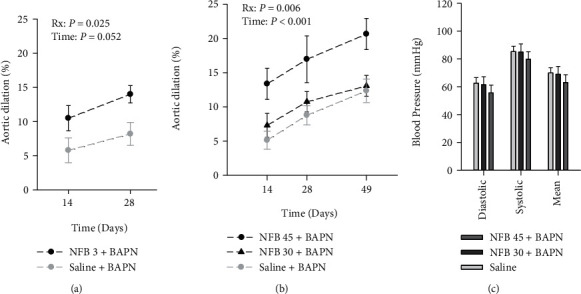
*β*-Aminopropionitrile (BAPN) sensitizes the response of TAADs to nicotine free base (NFB). (a, b) Effect of low (3.0 mg/kg/day, (a)) and high (30.0 or 45.0 mg/kg/day, (b)) doses of NFB on the growth of TAADs in mice given 0.2% BAPN; *n* = 5‐7 and *n* = 10‐17 per group for low- and high-dose experiments, respectively. (c) Blood pressure of mice exposed to high doses of NFB or saline on d49. Data were analyzed using two-way repeated measures ANOVA (a, b), or one-way ANOVA (c).

Although tobacco smoke is a well-established risk factor for TAAD and AAA development, it is a modulator rather than a driver. Aortic aneurysms do not develop in rodents exposed to cigarette smoke for half a year or longer [12]. It is possible that cigarette smoke could merely modulate the phenotype of TAADs in concert with certain pathogenic processes. We have previously shown that BAPN (*β*-aminopropionitrile) breaks the growth resistance of murine AAAs [23]. We reasoned that treatment with BAPN may improve the sensitivity of TAADs to nicotine. To test this hypothesis, animals (male, 11-16 weeks of age) were randomly assigned to BAPN in drinking water at concentrations of 0.2% (*n* = 7), 0.4% (*n* = 15), or 0.8% (*n* = 8), beginning three days before infusion of NFB at a dose of 3.0 mg/kg/day. This NFB dosage was chosen because of its success in modulating AAA formation [14, 15]. The control group (*n* = 5) received 0.2% BAPN in the drinking water and was implanted with saline-loaded pumps. All animals survived the 28-day follow-up period. Ultrasound imaging showed that over this 28-day period, mice treated with nicotine plus 0.2% BAPN incurred a significantly larger increase in TAAD diameter than those treated with 0.2% BAPN alone (14 ± 3% vs. 8 ± 2%, *P* = 0.021, Figure 2(a)). An increase in BAPN concentration was unable to further dilate the TAADs (*P* = 0.554, Figure S4a). Delivery of NFB at a rate of 3.0 mg/kg/day via osmotic minipumps resulted in constant concentrations of cotinine in the urine (Figure S4b) that were similar to those observed for smokers [20].

### 2.5. NFB Can Promote Aortic Rupture When Sensitized by BAPN

While successful in augmenting dilation of the TAADs, the challenge with 0.2% BAPN and NFB at a dose of 3.0 mg/kg/day was unable to induce aortic rupture, a fatal complication frequently occurring in TAAD patients [17]. Additionally, the small difference (~6%) in aortic dilation between NFB and saline-treated groups raised concerns about the statistical power for this model. We therefore examined whether an increase in NFB dosages is effective to boost aortic dilation and cause aortic rupture. Male mice (11-16 weeks of age) were randomly assigned to receive saline (*n* = 7) or NFB at dosages of 30.0 mg/kg/day (*n* = 10) or 45.0 mg/kg/day (*n* = 10) and followed with ultrasound imaging for 49 days. Administration of BAPN (0.2%) and tamoxifen followed the same timeline illustrated in Figure 1(a). Over the course of follow-up, one mouse in the group infused with 45.0 mg/kg/day NFB died from rupture of the ascending aorta on d12. Afterwards, aortic rupture or death was not observed. Animals receiving 30.0 mg/kg/day NFB all survived until the endpoint. By d49, mice challenged with 45.0 mg/kg/day NFB incurred a significantly greater TAAD dilation (21 ± 2%) than those receiving 30.0 mg/kg/day NFB (13 ± 2%, *P* = 0.027) or saline (12 ± 1%, *P* = 0.005, Figure 2(b)). The differences between groups treated with 30.0 mg/kg/day NFB or saline became insignificant after d28 due to “slow down” of the treated TAADs and “catch-up” of the control TAADs (Figure 2(b)). By d49, mice treated with either dose of NFB exhibited blood pressures similar to those obtained for the saline controls (Figure 2(c)) and gained a similar amount of body weight (2.9 ± 0.5 g and 3.8 ± 0.8 g for the groups treated with 30.0 mg NFB and 45.0 mg NFB, respectively; *P* = 0.323) compared to the saline controls (4.3 ± 0.6, *P* = 0.230), suggesting that the dosing regimens applied in this experiment are not systemically severely toxic nor do they cause significant changes in blood pressure.

### 2.6. Infusion of NFB Worsens Pathologies of the TAADs Induced by *Tgfbr2^iko^* at the Gross Level

In addition to aortic dilation, we evaluated the aortic pathology (periaortic adhesions, intramural hemorrhage, saccular aneurysm, and aortic rupture) via gross examination and histological analysis.

In the experiment where mice were treated with saline or nicotine salt (34.0 mg/kg/day), focal periaortic adhesions were noted in all TAADs of mice receiving saline or NS, with diffuse adhesions and intramural hemorrhage detected infrequently for both groups (Tables 1(a) and 1(b), Figure S5a). Similarly, differences in gross pathology were not evident in groups treated with NFB at 15.0 or 45.0 mg/kg/day or the saline control in the absence of BAPN (data not shown).

Treatment with NPs (5.0 mg, 90-day release, approximately 3.0 mg/kg/day) promoted diffuse periaortic adhesions in all survivors. In contrast, adhesions around placebo-treated TAADs were mostly focal (7/7 vs. 2/9, *P* = 0.003, Table 2(a)). Other pathologies, such as intramural hemorrhage and saccular aneurysms, were detected in the NP-treated but not in control mice (Tables 2(a) and 2(b)). These results together with the data of aortic dilation suggest that nicotine can exacerbate TAAD formation when administered at an effective dose.

In accordance with the similarity of aortic dilation among groups on various doses of BAPN, a further increase in the concentration of BAPN above 0.2% did not aggravate aortic damage in mice challenged with 3.0 mg/kg/day NFB (Table 3). Compared with the saline control, NFB failed to alter pathologies of the TAADs at the gross level when infused at a rate of 3.0 mg/kg/day (Table 3). In contrast, NFB infused at rates of 30.0 or 45.0 mg/day/kg promoted severe aneurysmal degeneration of the TAADs, as evidenced by the presence of diffuse periaortic adhesions and intramural hemorrhage, which were rarely detected under other conditions (Tables 1–4 and Figures 2(a)–2(c)). More strikingly, an aortic rupture, which never occurred in the control or low-dose NFB mice, occurred in the group challenged with 45.0 mg/kg/day NFB (Table 4(a)). Descending aortas, which nearly uniformly appeared grossly normal under other conditions, displayed diffuse adhesions, intramural hemorrhage, or saccular aneurysms in some mice challenged with NFB at dosages of 30.0 or 45.0 mg/kg/day NFB (Table 4(b)). These responses, however, were limited to the thoracic aorta because isolated AAAs were not found in these mice (Figure S5b).

### 2.7. Acceleration of TAAD Dilation by NFB Appears to Be Independent of Destruction of the Medial Architecture

**Figure 3:**
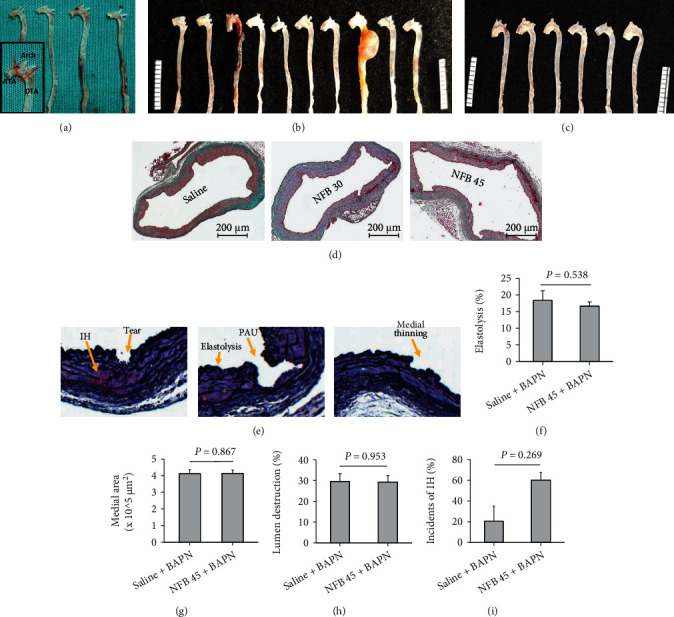
Nicotine free base (NFB) at doses sufficient to exaggerate aortic dilation may not worsen medial degeneration. (a–c) Gross appearance of TAADs in mice treated with saline ((a), *n* = 7) or NFB at dosages of 30.0 mg/kg/day (NFB 30, *n* = 10, (b)), or 45.0 mg/kg/day (NFB 45, *n* = 10, (c)). Ruler scale: mm. (d) Masson's staining of TAADs after undergoing the indicated treatments. Note the similarity in medial structural defects between these TAADs. (e) Typical pathologies disclosed with Movat's staining. IH: intramural hemorrhage; PAU: penetrating aortic ulcer. (f–i) Morphometric analyses in TAADs treated with saline (*n* = 10) or NFB (*n* = 6); (f) fractional area with elastolysis in the residual media; (g) cross-sectional area of the medial layer; (h) percentage of area with structural destruction on the luminal surface; and (i) incidence of IH.

Medial degeneration is a hallmark pathology underlying progression of TAAD development [17]. We examined whether the accelerated TAAD dilation in mice challenged with BAPN plus NFB results from exacerbation of medial architectural destruction. Because the treatment with BAPN +45.0 mg/kg/day NFB caused the largest amount of TAAD dilation (Figure 2(b)) and rupture, we focused our subsequent experiments on specimens generated with this protocol and compared them with control samples. Intimal/medial tears (defined as breaks of the intimal and medial layers with edges of the break being coarse and physically complementary), penetrating aortic ulcer (PAU, defined as an intimal/medial defect with an “erosion surface”), intramural hemorrhage (presence of red blood cells in the aortic wall), and medial thinning were detected in TAADs of either group (Figure 3(e)). Morphometric analysis was performed using the technique described in detail in Figure S6. The results showed that the fractional area with complete elastolysis, the cross-sectional area of the medial layer, the surface area with defects in structural integrity, and the incidence of fresh intramural hemorrhage were similar between the two groups (Figures 3(f)–3(i)), indicating that none of these structural defects is a dominant contributor to the differential TAAD dilation revealed by ultrasound imaging.

### 2.8. Treatment with NFB Results in Differential Effects on the Cellular Events Occurring in the Medial and Adventitial Layers of TAADs

**Figure 4:**
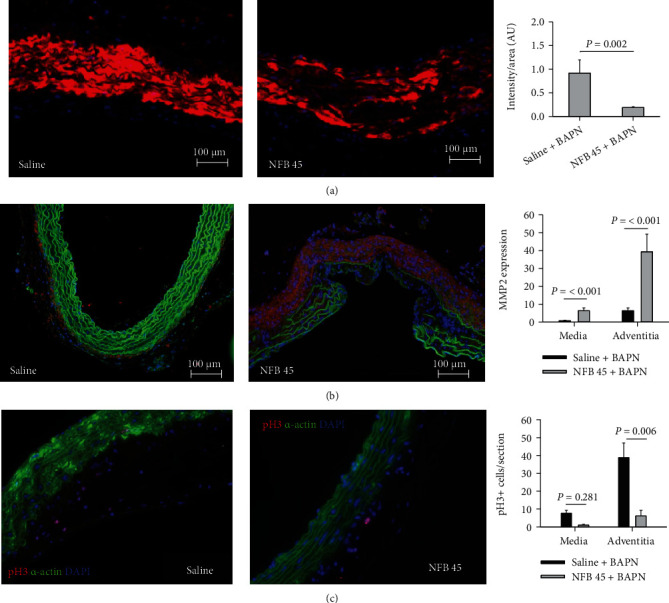
Chronic infusion of nicotine promotes phenotypic switching of smooth muscle cells to a synthetic phenotype in TAADs. (a–c) Assessment of production of *α*-actin (red, (a)), matrix metalloproteinase-2 (MMP-2, red, (b)), and phosphorylated histone 3 (pH 3, red, (c)) in TAADs, by IF staining (*n* = 5‐6 per condition). Blue: DAPI counterstain; green: autofluorescence (b) or *α*-actin (c). Measurements were normalized to the corresponding area and plotted in the bar graphs shown at the right. Data were analyzed using Student's *t*-test.

To study the mechanisms underlying the exacerbation of the TAAD dilation by NFB, we first evaluated the phenotype of SMCs. Immunofluorescence (IF) staining revealed significantly less production of *α*-actin in the medial layer of TAADs exposed to NFB as compared with the saline controls (*P* = 0.002, Figure 4(a)). NFB stimulated production of MMP2 in the TAADs, with the upregulation being much more pronounced in the adventitial layer (*P* < 0.001, Figure 4(b)). IF labeling of phosphorylated histone 3 (pH 3), a marker of cells in an active cell cycle state, showed that few cells in the medial layer of TAADs in either group had entered into the cell cycle (Figure 4(c)), indicating that the dedifferentiated SMCs were not proliferating in the TAADs. In contrast, proliferating cells were frequently observed in the adventitial layer, with NFB-treated TAADs showing significantly fewer proliferating cells than those in the saline control group (*P* = 0.006, Figure 4(c)).

### 2.9. Treatment with NFB Suppresses Adventitial Fibrosis in TAADs

**Figure 5:**
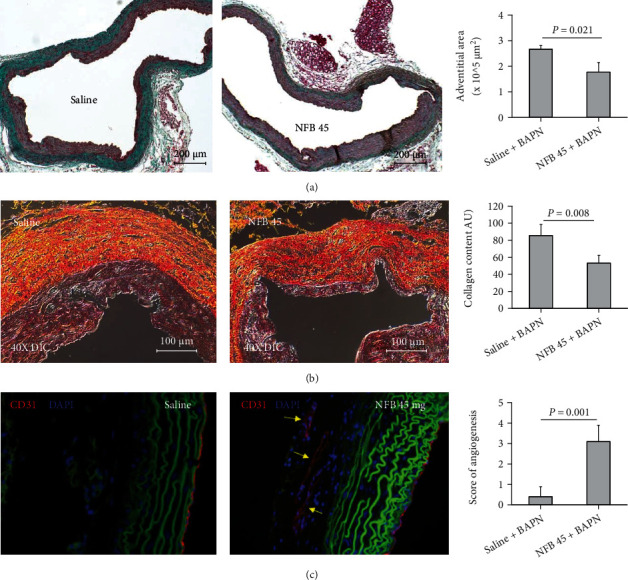
Chronic infusion of nicotine impairs TAAD adventitial fibrosis. (a) Masson's staining of TAADs treated with saline or nicotine free base (NFB 45). Area of the dense adventitia (green) was calculated by integrating *in vivo* diameter of the TAADs. (b) Picrosirius Red staining of TAADs illuminated with polarized light (i.e., differential interference contrast, DIC). Yellow and red substance represents collagens. The bar graph depicts collagen content in the adventitial layer of the TAADs. (c) IF labeling of CD31. Red: CD31+ structure; green: autofluorescence; blue: DAPI counterstain. Density of the microvessels in the adventitial layer was scored on a scale of 0 to 5. The bar graph shows scores assigned to the TAADs, with a larger score corresponding to a higher density. Data were analyzed using unpaired Student's *t*-test (*n* = 5‐7 per condition).

Microscopic evaluation of Masson-stained specimens noted a thinner adventitial layer in NFB-treated TAADs than in saline controls. This observation was further confirmed with morphometric analysis, which showed that NFB-treated TAADs were encompassed by significantly less fibrotic tissue compared with saline-treated TAADs (*P* = 0.021, Figure 5(a)). Next, we quantified deposition of collagen in the TAADs using Picrosirius Red and DIC (differential interference contrast) imaging. TAADs treated with NFB displayed significantly less accumulation of collagens than those treated with saline (*P* = 0.008, Figure 5(b)). Lastly, we evaluated angiogenesis, a process closely related to tissue fibrosis and aortic aneurysm development [24, 25], using IF staining of CD31. Floppy and dilated microvessels were frequently located in the adventitial layer of NFB-treated TAADs, whereas small CD31+ microvessels were scarcely scattered in the adventitial layer of TAADs of the saline control. Concordantly, NFB-treated TAADs displayed a significantly higher density of microvessels than their control counterparts in the adventitial layer (*P* = 0.001, Figure 5(c)).

### 2.10. Infusion of NFB Increases Infiltration of Inflammatory Cells in TAADs

**Figure 6:**
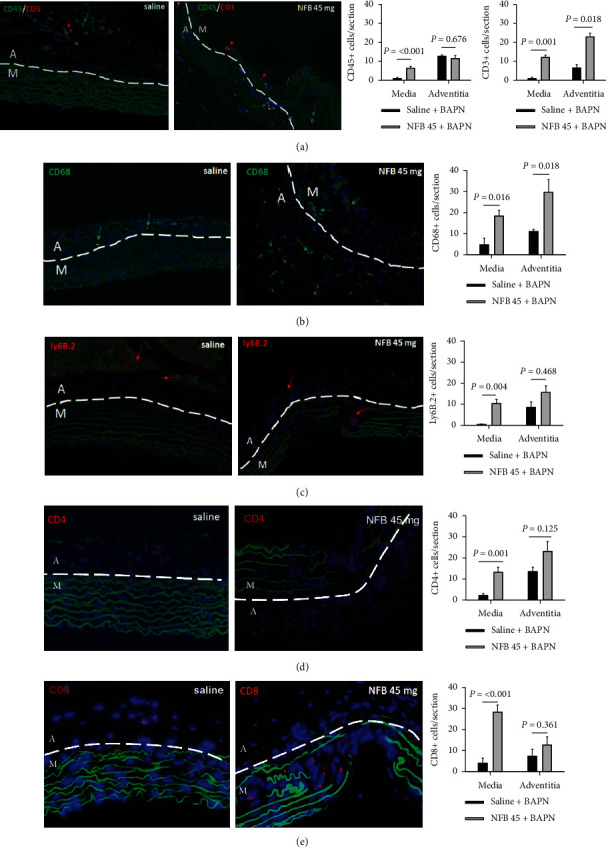
Treatment with nicotine free base (NFB) intensifies the chronic inflammation in TAADs. (a) Immunofluorescence (IF) double labeling of CD45+ leukocytes (green) and CD3+ T-cells (red). (b–e) IF labeling of macrophages (CD68+, green, (b)), neutrophils (Ly6B.2+, red, (c)), CD4+ T-cells (red, (d)), and CD8+ T-cells (red, (e)). Note that the inflammatory cells clustered largely in the area with structural destruction in NFB-treated TAADs, whereas in saline-treated TAADs, they were scattered sparsely across the aortic wall. A: adventitia; M: media; blue: DAPI counterstain. Data were analyzed using unpaired Student's *t*-test (*n* = 6‐9 per condition).

Infiltration of inflammatory cells was evaluated using IF assays. The staining revealed that leukocytes (CD45+, Figure 6(a)), 6T-cells (CD3+, Figure 6(a)), macrophages (CD68+, Figure 6(b)), and neutrophils (Ly6B.2+, Figure 6(c)) were sparsely scattered and located largely in the adventitial layer of TAADs of saline control mice. In contrast, quite a few leukocytes (CD45+), macrophages (CD68+), and CD3+ T-cells had infiltrated across the wall of TAADs of mice receiving NFB (Figures 6(a)–6(c)). Neutrophils (Ly6B.2+), helper T-cells (CD4+), and cytotoxic T-cells (CD8+) clustered in the vicinity of intimal/medial tears, PAUs, and dissections (Figures 6(c)–6(e)). Counts of these cells, normalized to the area in which the cells were located, showed that NFB-treated TAADs contained significantly more leukocytes, macrophages, neutrophils, and CD4+ or CD8+ T-cells than the saline controls in the medial layer (Figures 6(a)–6(e)). In the adventitia, however, such differences were observed only for macrophages and CD3+ T-cells (Figures 6(a) and 6(b)), while differences in other subsets were insignificant. This disparity in recruitment of inflammatory cells between the medial and adventitial layers is consistent with the compartmentalized effects of NFB on cellular events summarized in Figures 4 and 5 and indicates that local effects of NFB in the aortic wall are significant contributors to its detrimental consequence for TAAD formation.

## 3. Discussion

In the present study, we evaluated the impact of the chemical form, dose, delivery approach, and pathologic complexity on the efficacy of nicotine in exacerbating TAAD formation induced by *Tgfbr2^iko^*. The results demonstrated that (1) nicotine salt and free base, when delivered at doses usually applied in the fields of neuroscience and pulmonary disease, are not effective in modulating the phenotypic presentation of TAADs; (2) NFB by itself exacerbates TAAD formation only at doses approaching lethality; (3) treatment with BAPN overcomes the resistance of TAADs in response to NFB; (4) fueling local inflammation and impairing adventitial remodeling may represent critical processes by which NFB exacerbates TAAD formation.

Both nicotine salt and free base have been frequently administered in *in vivo* studies. The differential biological efficacy of these chemical forms was not recognized until a recent study demonstrating that the lipophilic free base form of nicotine is absorbed into the bloodstream at a much higher rate than the hydrophilic nicotine salt [26]. This finding indicates that, in organs such as the aorta, the bioavailability of a given dose of the nicotine ligand to its nicotinic acetylcholine receptors (nAChRs) would be much lower when delivered in salt form rather than as free base. In tobacco leaves, nicotine is mostly protonated. However, when heated to >200°C in a burning cigarette or volatizing pod, the protonated nicotine undergoes thermal transfer to unprotonated nicotine in the gas phase [27]. This thermal transfer occurs during smoking but may not happen in implanted pumps where the temperature is far below the transferring point. Although the monoprotonated nicotine binds to nAChRs at a much greater affinity than the unprotonated form [28], bioavailability of nicotine in the target organ appears to be a critical factor to consider when choosing a chemical form for *in vivo* studies. The differential diffusion rates of nicotine salt vs. free base across the aortic wall might also have contributed to the minimum impact of nicotine salt on TAAD formation observed in the present study.

In reported studies in which NFB was administered to mice, the dosage has varied widely. In studies with objectives focusing on addiction, the drug was often infused for weeks via venous catheters or osmotic pumps at rates ranging from <10.0 mg/kg/day [20] to 48.0 mg/kg/day or higher [29–31]. In the fields of cancer, psychiatry, and pulmonary disease research, the drug was often administered at a dosage <10.0 mg/kg/day [20]. Few studies have reported successful application of NFB to modulate phenotypic expression of AAAs. They show that NFB delivered at a relatively low dosage (<5.0 mg/kg/day) resulted in remarkable exacerbation of AAAs induced by elastase or AngII [14, 15]. Similarly, tobacco smoke exacerbated elastase-induced AAA formation in mice [9] when delivered at a dose resulting in serum cotinine concentrations (106.0 ng/ml) within the lower range observed among smokers (~400.0 ng/ml) [20, 32]. Furthermore, Wang et al. showed that NFB delivered at a dosage of 1.0 mg/kg/day induced AAA formation in naïve ApoE-/- mice [15]. It is obvious that in the literature, the dosage of NFB administered to mice varies among studies in the literature, depending on the specific pathologic conditions. Consistent with this notion, the present study showed that NFB alone failed to impact TAAD formation until it approached to the lethal dose. However, when a “second hit” (i.e., BAPN, an inhibitor of lysyl oxidase) was introduced to alter the baseline pathology, NFB augmented dilation of the TAADs at a fairly modest dosage (3.0 mg/kg/day) and promoted further dilation and aortic rupture when it was increased to 45.0 mg/kg/day. It is noteworthy that this high dose of NFB (i.e., 45.0 mg/kg/day) was well-tolerated by mice, as evidenced by the absence of rupture-unrelated death and by weight gains similar to the saline controls during the seven-week follow-up period. Aortic rupture observed in NFB-treated mice most likely results from its effect on aneurysm degeneration rather than hypertension because blood pressure in these mice was not significantly different from their control counterparts. Another noteworthy point to emphasize is that none of the mice enrolled in the present study displayed significant dilation or gross pathology of the abdominal aorta, even when challenged with BAPN plus the highest dosage of NFB. This observation appears to be conflict with the finding reported by Wang et al. showing that NFB not only exacerbates but also causes AAA formation in ApoE-/- mice [15]. It is known that AAAs and TAADs may proceed via differential cellular and molecular mechanisms [17, 33, 34]. We believe that the potential controversy between AAA-focused studies and the present study simply echoes the concept that various pathological processes contextually respond to different minimum effective doses of NFB.

Nicotine is a significant modulator of several biological processes such as immune responses [35], angiogenesis [36], and tissue remodeling [37]. In the present study, we showed that macrophages, neutrophils, and T-cells were located in the area of intimal/medial tears, PAUs, and dissections in TAADs treated with NFB, with only a few of these cells present in TAADs of control mice receiving the saline control. These results suggest that infusion of NFB increases the chronic inflammation that otherwise would persist at a mild intensity. Previous studies from other groups have suggested that nicotine may facilitate the transfer of the innate immune response to adaptive immunity in aortic aneurysms [9, 38]. In echoing these findings, we identified significantly more T-cells, particularly helper (CD4+) and cytotoxic (CD8+) T-cells, in the medial layer of TAADs treated with NFB than with saline. Treatment with NFB causes production of higher levels of MMP2 and more rigorous neovascularization in the adventitial layer of TAADs, compared with saline-treated controls. These observations are consistent with other studies focusing on AAAs [14, 15, 24, 39] and TAADs [25] and point toward roles of nicotine in regulation and propagation of the local inflammatory response, leading to exacerbation of TAAD formation. Other mechanisms, such as epigenetic regulation [40] and chemical modification of matrix components especially elastin fibers [41], have been implicated in the detrimental effects of nicotine on tissue homeostasis and might be applicable in the context of aortic aneurysm formation.

An intriguing finding made in the present study is that chronic infusion of NFB may result in weakening of the adventitia compared to saline controls. The adventitia provides the ultimate strength to the aortic wall. Weakening of the mechanical strength of this layer has been postulated as a critical pathway leading to progressive aortic dilation and rupture [42]. We have reported that the protective effects of estrogen against TAAD formation are associated with thickening of the adventitial layer [43]. Consistent with this observation, the present study demonstrated that the detrimental effects of NFB on TAAD formation correlated with an attenuated adventitial thickening. Recently, Kawamura et al. showed that in aortas deficient in SMC-specific TGF*β* signaling, compensatory adventitial thickening preserves the mechanical strength of the aortic wall, reducing the risk of aortic rupture [44]. It appears that adventitial remodeling is the common pathway through which risk factors, such as sex and smoking, modulate development of aortic aneurysms. Adventitial remodeling is a sophisticated process that involves complex cellular and molecular events [45]. Results of the present study suggest that attenuated cell proliferation, elevated MMP2 production, exaggerated inflammation, and pathogenic angiogenesis might be responsible for the blunted adventitial fibrosis in NFB-treated TAADs.

The incidence of aortic rupture in NFB-treated mice is quite low when challenged with the protocol performed in the present study, indicating that there remains room to increase the dose of NFB. Mice are known to be much less sensitive to nicotine than other species and are able to metabolize 50% of serum nicotine in as few as 6 minutes [20]. Other studies showed that mice can be safely dosed with NFB at a rate up to 144.0 mg/kg/day [29, 30]. A further increase in the drug dose may increase the rate of aortic rupture, thus, improving the model's performance. A caveat for administering NFB to adult mice at a rate > 10.0 mg/kg/day is that it may elevate the level of serum nicotine above 100.0 ng/ml, which is higher than the peak arterial nicotine concentration detected in human smokers [20]. However, the disparity in dose-response between different species appears not to be uncommon. For instance, losartan must be administered to mice at a dosage > 100 times higher than that prescribed for humans to achieve similar effects on blood pressure [46, 47]. Another limitation of the present model is the requirement for sensitization with BAPN. While BAPN might interfere with the intrinsic tissue repairing process, thus making aneurysms more vulnerable to nicotine, further studies are warranted to determine whether the model continues to require BAPN when challenged with cigarette smoke, which contains thousands of components other than nicotine [48]. Wang et al. reported that a relatively low dose of NFB (1.0 mg/kg/day) alone promoted AAA formation in mice infused with AngII [15], which contrasts to our finding that a much higher dose of NFB (i.e., 45.0 mg/kg/day) was unable to affect TAAD formation. While the exact cause for this disparity was not determined in the present study, we suspect that differences in model creation and anatomic location are the major outcome determinants for an aortic aneurysm following exposure to nicotine. Additionally, the age of mice in this study varied from 9 to 18 weeks. Although neither aortic diameter nor aortic dilation appeared to vary in an age-dependent fashion, it is not statistically sound to rule out potential contribution of age due to insufficient sample size. Finally, our results suggest that the detrimental effects of nicotine on TAAD formation are associated with intensified inflammation and attenuated adventitial fibrosis. This is, by no means, meant to be comprehensive. Subunit-specific nAChR signaling, stimulation of protease production, modulation of immune cell differentiation, cell toxicity, etc., have all been implicated as potential mechanisms responsible for the cardiovascular effects of nicotine [12, 35, 49, 50]. These mechanisms may play an important role in nicotine exacerbation of TAADs and should be evaluated in future studies.

In summary, thoracic aortic aneurysms and dissections proceed via complex biological processes that may be difficult to recapitulate with a single animal model. Nicotine, one of the major components of tobacco smoke, can modulate only some of these processes, with the effective dose varying according to the specific target process (i.e., dilation vs. rupture). Under conditions optimized in the present study, enhanced chronic inflammation and suppression of adventitial thickening emerged as prominent mechanisms through which nicotine modulates dilation and rupture of TAADs.

## 4. Materials and Methods

### 4.1. Animals

This study conforms to the Guide for the Care and Use of Laboratory Animals of the National Institutes of Health. All the protocols were approved by the Institutional Animal Care and Use Committee of the University of Florida. An inducible Cre-loxP system, driven by the myosin heavy chain 11 (Myh11) promoter, was used for mutant construction. Briefly, the Tgfbr2^f/f^ (Tgfbr2-floxed) strain was crossed with a Y-linked Myh11-CreER strain. Offspring litters were screened with PCR-based genotyping assays to establish a colony with a *Tgfbr2^f/f^Myh11-CreER^+^* genotype. The ancestor strains for this colony were backcrossed to C57BL/6J background for >5 generations [18].

Tamoxifen (Sigma, T5648, 2.5 mg/mouse) was dissolved in corn oil (25.0 mg/ml, Sigma, C8267) and administered via daily *i.p.* injections for five consecutive days prior to treatment with smoke mimetics at the indicated chemical form and dosage. In experiments involving *β*-aminopropionitrile (BAPN), the drug (Sigma, A3134) was delivered via drinking water beginning three days before tamoxifen injection. Osmotic minipumps (Alzet 2004, Cupertino, CA) were implanted as previously described [51]. Briefly, pumps loaded with nicotine solution were primed by incubating in saline (37°C) overnight. Animals were anesthetized via inhalation of 1.0-1.5% isoflurane. Then, a transverse incision (~6 mm) was made on the back slightly below the right shoulder. A curved tissue dissection scissor was inserted through the incision to make a subcutaneous tunnel similar in a dimension to the pump, followed by inserting the pump into the tunnel and closing the incision with metal clips. Cigarette smoking extract (CSE) was made by bubbling smoke of nine cigarettes (3R4F, Kentucky Tobacco Research & Development Center, Lexington, KY) through 3 ml PBS. CSE was injected *i.p.* daily, at a dose of 0.1 ml per mouse [22, 52]. The day of the first tamoxifen injection was considered to be day 0. Aortic samples were surgically harvested for morphological analysis.

### 4.2. Gross Examination

All aortas were grossly evaluated under an operating scope by a blinded observer for periaortic adhesions (none, focal, or defused), intramural hemorrhage (presence of yellow wall, red dots/patches, or clot), saccular aneurysms, and rupture (presence of hemothorax or hemoabdomen).

### 4.3. Ultrasound Imaging

Animals were anesthetized via inhalation of 1.5-2.0% isoflurane and placed in a supine position on a heated platform, followed by removing the fur in the area of imaging with a depilatory cream and warm water. A high-resolution Vevo 2100 Imaging System with an MS550D (central frequency: 40 MHz) linear array transducer (VisualSonics) was then utilized to scan aortas under B-mode as we have described in detail previously [18, 53]. Briefly, TAADs and AAAs were imaged via the long axis view. A cineloop was then reviewed to select a frame for measurement of the lumen diameter of the aorta. For TAADs, a straight line was drawn from the point of 11 o'clock of the right pulmonary artery to the opposite wall with the line adjusted to such a position that it symmetrically divided the trunk of the ascending aorta. For AAAs, the lumen diameter was measured at the point approximately 2.0 mm proximal to the celiac artery. Aortic dilation was calculated using the following functions:

(1) $$\begin{array}{c} \text{Aortic dilation }\left(\%\right)=\left(\frac{D_{1}-D_{0}}{D_{0}}\right)\times 100, \end{array}$$

where *D*_0_ and *D*_1_ indicate aortic diameter estimated with ultrasound imaging at d0 and on the day of endpoint assessment, respectively.

### 4.4. Histology

All aortas were perfusion-fixed with 10% neutral buffered formalin during sample collection. A series of paraffin-embedded cross-sections (5.0 *μ*m) were collected at positions 0 (P0), 100 (P100), and 200 (P200) *μ*m from the proximal end of TAADs. The P0 was defined as the point at which a full-circle cross-section was first observed during the sectioning. Multiple sets of slides were collected for each location and used for Masson's, Movat's, and immunohistochemistry/IF staining.

### 4.5. Morphometric Analysis

The short length of mouse TAADs makes it technically difficult to prepare cross-sections at an angle perfectly perpendicular to the long axis of the aortic segment. As the result, the cross-sectional areas obtained for those sections often deviate unacceptably far away from their true values. To overcome this difficulty, we used medial thickness obtained from cross-sections and *in vivo* diameter, measured with ultrasound imaging, to calculate the medial area for comparisons between groups treated with and without smoke mimetics. The assumption is that cross-sections of a fixed straight tube, such as a perfusion-fixed aorta, may vary significantly in cross-sectional area, but to a less degree in wall thickness.

Morphological analysis was performed on cross-sections using ZEN lite 2012 (Zeiss), calculating an average of the measurements obtained at P0, P100, and P200 to represent that sample. The following functions were utilized to estimate the cross-sectional area of the medial layer of TAADs:

(2) $$\begin{array}{c} \text{Medial thickness}=\frac{A_{\text{EEL}}-A_{\text{IEL}}}{{\text{CL}}_{\text{EEL}}},\\ \text{Medial area}=\text{medical thickness}\times D_{\text{UL}}\times \pi , \end{array}$$

where *A*_EEL_, *A*_IEL_, CL_EEL_, and *D*_UL_ indicate area within the external elastic lamina (EEL), area within the internal elastic lamina (IEL), circumferential length of the EEL, and diameter measured with ultrasound imaging, respectively.

Lengths of the luminal surface breaks and areas with elastolysis were measured on cross-sections and normalized to the length of EEL and the area of media, respectively, using the ZEN Blue analysis software (Zeiss). Specifically, luminal surface breaks were traced individually, summed, and normalized to the length of EEL of the analyzed section (Figure 6(a)). Areas with complete elastolysis (Figure S6b) were detected and selected with the Advanced Interactive Measurement module. Filters were applied to exclude “physiological space” between elastic laminae. All analyzed images were reviewed individually, with the filter settings adjusted to ensure specific selection of the objects of interest.

### 4.6. Immunofluorescence Staining

All assays were performed on formalin-fixed, paraffin-embedded sections. Antigens were unmasked by incubating specimens in citrate buffer (pH 6.0, Vector Lab, H-330), heated and pressurized with a pressure cooker. After blocking of nonspecific bindings, specimens were incubated with primary antibodies at 4°C overnight, followed by labeling with Alexa Fluor-conjugated secondary antibodies (Table 5) at room temperature for 1 hour. Nuclei were counterstained with DAPI (Sigma, D9542). Information for all antibodies is provided in Table 5. All samples were imaged with a monochrome camera operated by ZEN lite 2012 (Zeiss). Total intensity of *α*-actin staining was normalized to the area of media, whereas counts for inflammatory cells, pH 3-positive cells, and CD31+ structures were normalized to the area in which the positive cells were located.

### 4.7. Picrosirius Red Staining

Paraffin-embedded cross-sections (5.0 *μ*m) were deparaffinized and rehydrated. Picrosirius Red solution (Abcam, ab150681) was applied to completely cover sections and incubated for 1 hour. Sections were rinsed in two changes of acetic acid solution, two changes of absolute alcohol, and one change of xylene, followed by mounting with coverslips. The staining was illuminated under a setting for differential interference contrast (DIC) imaging and recorded with a color camera. Images were analyzed using the interactive measurement module of ZEN lite 2012 (Zeiss). Briefly, a threshold was set for red, green, and blue channels. Then, the set threshold was applied to select and quantify areas with “positive” staining in the adventitial layer. The area of “positive” staining, normalized to area of the adventitia, was taken to represent collagen content in that sample.

### 4.8. Statistical Analysis

All data are expressed as the mean ± SEM. Statistical analyses were performed using Sigma Plot 14.0 (San Jose, CA, USA). Datasets were evaluated using normality and equivalence variance testing. For those failing this evaluation, logarithmic and exponential transformations were performed to meet these requirements. Student's *t*-test, one-way ANOVA, two-way ANOVA, and two-way repeated measures ANOVA were performed, when appropriate, with Holm–Sidak analysis being used for post hoc tests. *P* < 0.05 was considered statistically significant.

## Figures and Tables

**Table:** (a) Pathology in ascending aortas

Rx	Adhesion		Intramural hemorrhage	Saccular aneurysm	Aortic rupture
	Focal	Diffuse			
Saline (*n* = 8)	8	0	0	0	0
Nicotine salt (*n* = 8)	8	0	1	0	0

**Table:** (b) Pathology in descending aortas

Rx	Adhesion		Intramural hemorrhage	Saccular aneurysm	Aortic rupture
	Focal	Diffuse			
Saline (*n* = 8)	0	1	0	0	0
Nicotine salt (*n* = 8)	0	0	0	0	0

Note: statistical analysis was not performed due to rare frequency of the expressed phenotypic traits in either group.

**Table:** (a) Pathology detected in ascending aortas

Rx	Adhesion		Intramural hemorrhage	Saccular aneurysm	Aortic rupture
	Focal	Diffuse			
Placebo (*n* = 9)	9	2	0	0	0
Nicotine pellet (*n* = 7)	7	7^∗^	1	0	0

**Table:** (b) Pathology in descending aortas

Rx	Adhesion		Intramural hemorrhage	Saccular aneurysm	Aortic rupture
	Focal	Diffuse			
Placebo (*n* = 9)	0	1	0	0	0
Nicotine pellet (*n* = 7)	0	2	1	1	0

^∗^ *P* = 0.003, Fisher exact test.

**Table 3:** Gross pathology detected in the ascending aorta of mice receiving saline or NFB (3.0 mg/kg/day) plus BAPN.

Rx	Adhesion		Intramural hemorrhage	Saccular aneurysm	Aortic rupture
	Focal	Defused			
Saline+0.2% BAPN (*n* = 5)	5	2	0	0	0
NFB+0.2% BAPN (*n* = 7)	7	4	0	0	0
NFB+0.4% BAPN (*n* = 15)	15	7	0	0	0
NFB+0.8% BAPN (*n* = 8)	8	7	1	0	0

Note: pathology was not detected in descending aortas.

**Table:** (a) Pathology detected in ascending aortas

Rx	Adhesion		Intramural hemorrhage	Saccular aneurysm	Aortic rupture
	Focal	Defused			
Saline (*n* = 7)	3	4	0	0	0
NFB 30 (*n* = 10)	0	10^∗^	3	0	0
NFB 45 (*n* = 10)	0	7	1	0	1

^∗^ *P* = 0.051, Fisher exact test.

**Table:** (b) Pathology detected in descending aortas

Rx	Adhesion		Intramural hemorrhage	Saccular aneurysm	Aortic rupture
	Focal	Defused			
Saline (*n* = 7)	6	0	0	0	0
NFB 30 (*n* = 10)	0	2	1	1	0
NFB 45 (*n* = 10)	0	2	2	0	0

**Table 5:** Information of antibodies used in this study.

Antigen	Antibody	Vender	Titer
CD45	Rat anti-mouse IgG2	30-F11, BD Pharm. Inc, San Diego, CA	1 : 200
CD3	Rabbit anti-mouse IgG	Ab5690, Abcam, Waltham, MA	1 : 200
Ly6b.2	Rat anti-mouse IgG2	MCA771G, Bio-Rad, Hercules, CA	1 : 100
CD68	Rabbit anti-mouse IgG	PA5-78996, ThermoFisher, Waltham, MA	1 : 250
CD4	Rabbit anti-mouse IgG	MAB114-100, ThermoFisher, Waltham, MA	1 : 50
CD8	Rabbit anti-mouse IgG	MA5-14548, ThermoFisher, Waltham, MA	1 : 50
*α*-Actin	Cy3-mouse IgG	C6198, Sigma-Aldrich, St. Louis, MO	
Rabbit IgG	Alexa Fluor 488 goat IgG	A11008, Life Technologies, Carlsbad, CA	1 : 200
Rat IgG	Alexa Fluor 546 goat IgG	A11081, ThermoFisher, Waltham, MA	1 : 200
Rat IgG	Alexa Fluor 488 goat IgG	A11006, ThermoFisher, Waltham, MA	1 : 200
Phospho-histone 3 (Ser10)	Rabbit anti-mouse	PA5-85506, Invitrogen, Waltham, MA	1 : 500
CD31	Rat anti-mouse IgG2a, k	557355, BD Biosciences, San Diego, CA	1 : 250
MMP2	Goat anti-mouse/rat IgG	AF1488, R&D Systems, Minneapolis, MN	1 : 50

## Data Availability

Additional data (figures) used to support the findings of this study are included within the supplementary information file(s).

## Additional Points

*New and Noteworthy*. TAADs created in animal models may not be readily responsive to the insult of tobacco smoke mimetics. Enhancement of the pathologic complexity of the TAADs with BAPN sensitizes their responsiveness to NFB, with increased aortic ruptures being induced by increasing NFB dosages. Infusion of NFB fuels chronic inflammation in TAADs and drives maladaptive remodeling of the adventitia.

## Supplementary Materials

Supplementary Materials Figure S1: nicotine salt failed to affect aortic dilation when delivered at a rate of 34.0 mg/kg/day (equivalent to 11.1 mg/kg/day of nicotine free base). (a) Aortic dilation of TAADs exposed to nicotine salt (*n* = 10) or saline (*n* = 9). Data were analyzed using two-way repeated measures ANOVA. (b) Representative ultrasound scanning images of mice undergoing the indicated treatments for 42 days. Green lines illustrate the plane in which the diameter of the TAAD was measured. Figure S2: the detrimental effects of nicotine pellets on aortic dilation were not associated with severe general toxicity nor changes in blood pressure. (a) Changes in body weight during the 42-day follow-up period. (b) Blood pressure of mice treated with pellets containing nicotine free base (NFB) or placebo for 42 days. The differences in systolic, diastolic, and mean blood pressure were not significant between groups receiving nicotine pellets (NPs) and placebo, as analyzed using unpaired Student's *t*-test. (c, d) Relationships between age and diameter of the ascending aorta prior to pellet implantation (C) or aortic dilation estimated at d42 for mice that survived the initial burst of NFB release (D). Figure S3: an extract of cigarette smoke (CSE) was unable to augment dilation of TAADs. Mice treated with CSE (*n* = 9) or PBS (*n* = 6) presented similar amount of aortic dilation during the 28-day follow-up period. Data were analyzed using two-way repeated measures ANOVA. Figure S4: an increase in the concentration of *β*-aminopropionitrile (BAPN) to higher than 0.2% in drinking water did not impact aortic dilation to a greater degree. (a) Aortic dilation over 28 days. (b) Cotinine concentration in urine collected from mice receiving saline or nicotine free base (NFB, 3.0 mg/kg/day). Data were analyzed using one-way ANOVA. Figure S5: gross aorta specimens from mice treated with various protocols. (a) Aortas of mice treated with saline or nicotine salt for 42 days. (b) Aortas of mice treated with 30.0 or 45.0 mg/kg/day nicotine free base (NFB) plus 0.2% *β*-aminopropionitrile (BAPN) for 49 days. Note that all abdominal aortas appeared grossly normal unless affected by a dissected thoracic aorta. Scale of rulers is in mm. Figure S6: illustration of them morphometric analysis. (a) Quantification of % surface area with defects in structural integrity, including intimal/medial tears, penetrating aortic ulcer, and medial thinning. Blue line: external elastic lamina; red line: width of the area losing intimal/medial layers. (b) Measurements performed to estimate % cross-sectional area with elastic fiber breaks. Green line: outline of the residual medial layer; red objects: areas losing elastic fibers. Note the accurate fill of areas lacking elastic fibers (arrows) by the red objects in the magnified views.
