# Supplementary material for: Nicotine Exacerbates TAAD Formation Induced by Smooth Muscle-Specific Deletion of the TGF-β Receptor 2
Source: J Immunol Res. 2021 Oct 1;2021:6880036. doi: 10.1155/2021/6880036 (PMC8505064; doi:10.1155/2021/6880036)
Supplement: Supplementary Materials — Figure S1: nicotine salt failed to affect aortic dilation when delivered at a rate of 34.0 mg/kg/day (equivalent to 11.1 mg/kg/day of nicotine free base). (a) Aortic dilation of TAADs exposed to nicotine salt (n = 10) or saline (n = 9). Data were analyzed using two-way repeated measures ANOVA. (b) Representative ultrasound scanning images of mice undergoing the indicated treatments for 42 days. Green lines illustrate the plane in which the diameter of the TAAD was measured. Figure S2: the detrimental effects of nicotine pellets on aortic dilation were not associated with severe general toxicity nor changes in blood pressure. (a) Changes in body weight during the 42-day follow-up period. (b) Blood pressure of mice treated with pellets containing nicotine free base (NFB) or placebo for 42 days. The differences in systolic, diastolic, and mean blood pressure were not significant between groups receiving nicotine pellets (NPs) and placebo, as analyzed using unpaired Student's t-test. (c, d) Relationships between age and diameter of the ascending aorta prior to pellet implantation (C) or aortic dilation estimated at d42 for mice that survived the initial burst of NFB release (D). Figure S3: an extract of cigarette smoke (CSE) was unable to augment dilation of TAADs. Mice treated with CSE (n = 9) or PBS (n = 6) presented similar amount of aortic dilation during the 28-day follow-up period. Data were analyzed using two-way repeated measures ANOVA. Figure S4: an increase in the concentration of β-aminopropionitrile (BAPN) to higher than 0.2% in drinking water did not impact aortic dilation to a greater degree. (a) Aortic dilation over 28 days. (b) Cotinine concentration in urine collected from mice receiving saline or nicotine free base (NFB, 3.0 mg/kg/day). Data were analyzed using one-way ANOVA. Figure S5: gross aorta specimens from mice treated with various protocols. (a) Aortas of mice treated with saline or nicotine salt for 42 days. (b) Aortas of mice treated with 30. [file 6880036.f1.pdf]

Supplemental data

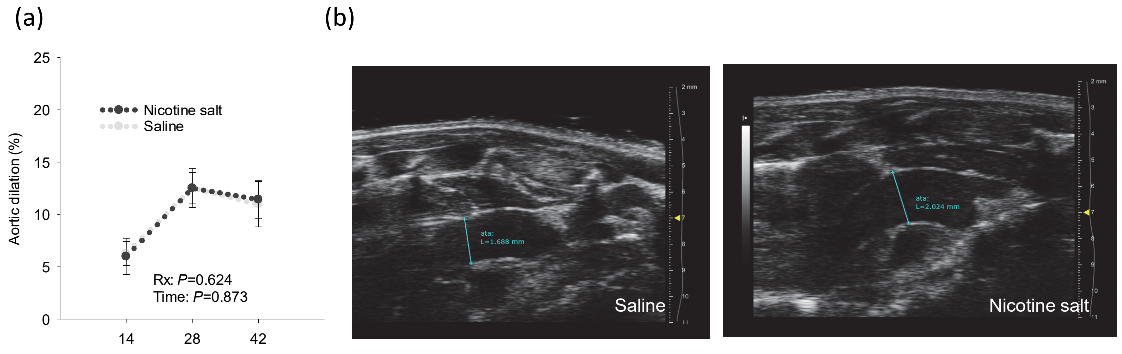

Figure S1. Nicotine salt failed to affect aortic dilation when delivered at a rate of 34.0 mg/kg/day (equivalent to 11.1 mg/kg/day of nicotine free base). (a), Aortic dilation of TAADs exposed to nicotine salt (n=10) or saline (n=9). Data were analyzed using two-way repeated measures ANOVA. (b), Representative ultrasound scanning images of mice undergoing the indicated treatments for 42 days. Green lines illustrate the plane in which diameter of the TAAD was measured.

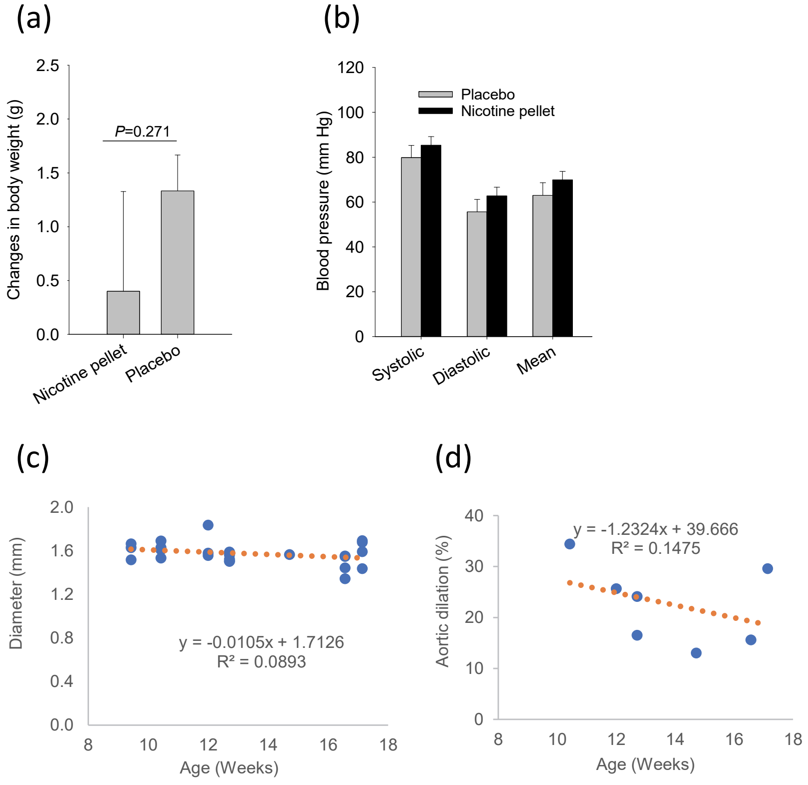

Figure S2. The detrimental effects of nicotine pellets on aortic dilation were not associated with severe general toxicity nor changes in blood pressure. (a), Changes in body weight in during the 42-day follow-up period. (b), Blood pressure of mice treated with pellets containing nicotine free base (NFB) or placebo for

42 days. The differences in systolic, diastolic, and mean blood pressure were not significant between groups receiving nicotine pellets (NPs) and placebo, as analyzed using unpaired Student's t-test. (c) and (d), Relationships between age and diameter of the ascending aorta prior to pellet implantation (C) or aortic dilation estimated at d42 for mice that survived the initial burst of NFB release (D).

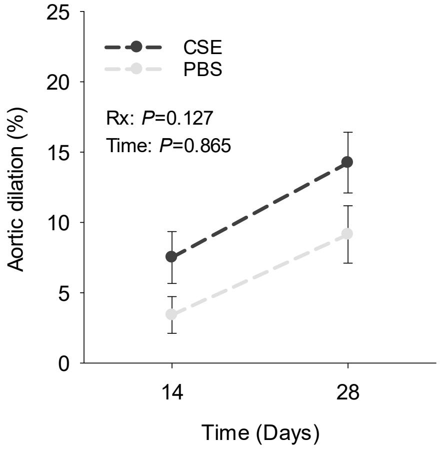

Figure S3. An extract of cigarette smoke (CSE) was unable to augment dilation of TAADs. Mice treated with CSE (n=9) or PBS (n=6) presented similar amount of aortic dilation during the 28-day follow-up period. Data were analyzed using two-way repeated measures ANOVA.

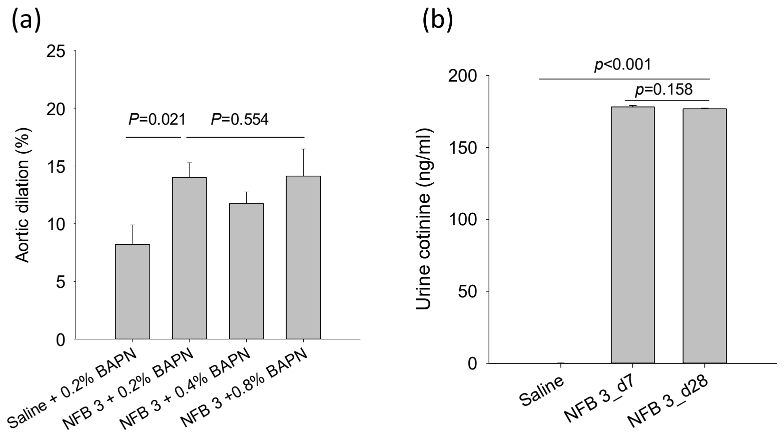

Figure S4. An increase in the concentration of β-aminopropionitrile (BAPN) to higher than 0.2% in drinking water did not impact aortic dilation to a greater degree. (a), Aortic dilation over 28 days. (b), Cotinine concentration in urine collected from mice receiving saline or nicotine free base (NFB, 3.0 mg/kg/day). Data were analyzed using one-way ANOVA.

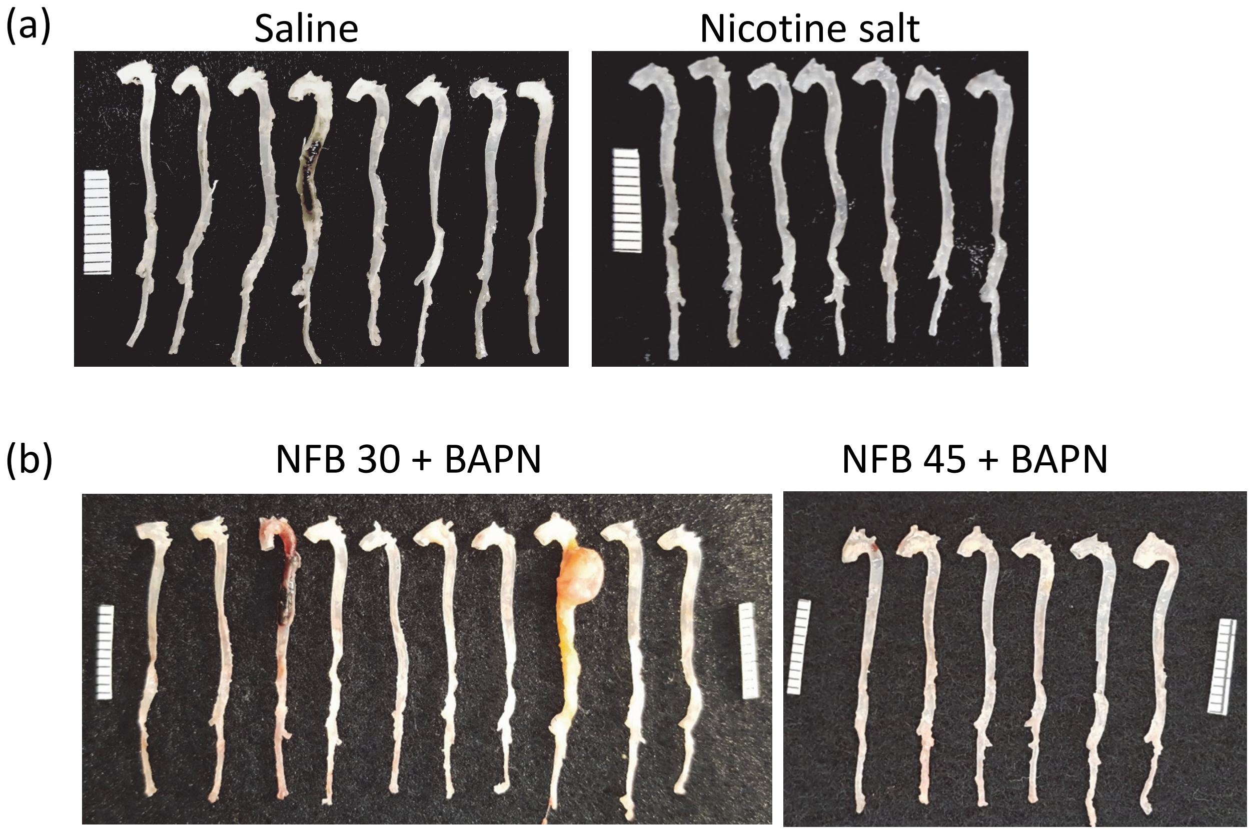

Figure S5. Gross aorta specimens from mice treated with various protocols. (a), Aortas of mice treated with saline or nicotine salt for 42 days. (b), Aortas of mice treated with 30.0 or 45.0 mg/kg/day nicotine free base (NFB) plus 0.2% β-aminopropionitrile (BAPN) for 49 days. Note that all abdominal aortas appeared grossly normal unless affected by a dissected thoracic aorta. Scale of rulers are in mm.

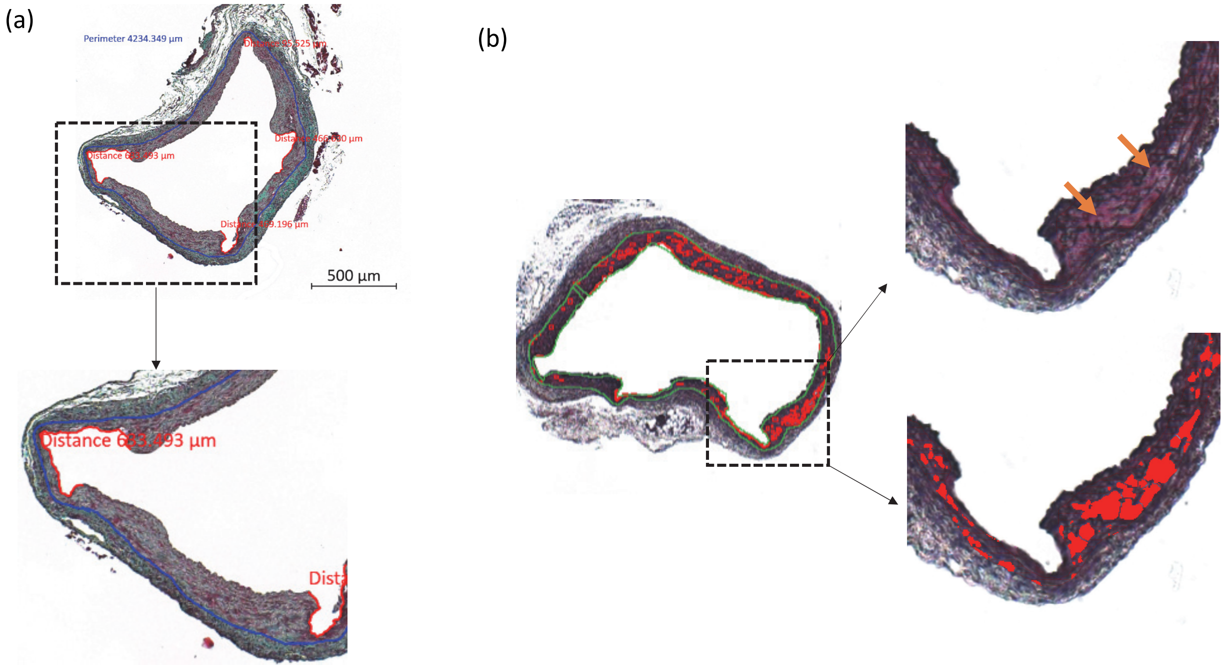

Figure S6. Illustration of them morphometric analysis. (a), Quantification of % surface area with defects in structural integrity, including intimal/medial tears, penetrating aortic ulcer, and medial thinning. Blue line: external elastic lamina; red line: width of the area losing intimal/medial layers. (b), Measurements performed to estimate % cross-sectional area with elastic fiber breaks. Green line: outline of the residual medial layer; red objects: areas losing elastic fibers. Note the accurate fill of areas lacking elastic fibers (arrows) by the red objects in the magnified views.
